# Association between body image satisfaction and depressive symptoms among young women in the UAE with Polycystic Ovary Syndrome (PCOS) and Non-PCOS: A comparative quantitative cross-sectional study

**DOI:** 10.1371/journal.pone.0333908

**Published:** 2026-01-06

**Authors:** Shaima Abdrahman AlMekhlafi, Maria Susana Campo-Redondo

**Affiliations:** 1 Department of Clinical Psychology, College of Medicine & Health Sciences, United Arab Emirates University, Al Ain, United Arab Emirates; 2 Maudsley Health, Abu Dhabi, United Arab Emirates; UFRN: Universidade Federal do Rio Grande do Norte, BRAZIL

## Abstract

**Background:**

Polycystic Ovary Syndrome (PCOS) is one of the most common conditions affecting women of reproductive age, significantly impacting both physical and psychological well-being, often leading to depressive symptoms and lower body image satisfaction. The prevalence of Polycystic Ovary Syndrome among women in the United Arab Emirates (UAE) is increasing. However, the psychological effects of Polycystic Ovary Syndrome on women in the UAE remain underexplored due to cultural sensitivities surrounding reproductive health. This cross-sectional study examines the association between depressive symptoms and body image satisfaction among young women in the UAE, comparing those with and without Polycystic Ovary Syndrome. The study surveyed 221 women aged 18–30, with 94 having Polycystic Ovary Syndrome and 127 without. Data were collected using the Patient Health Questionnaire-9 (PHQ-9), Body Image Concern Inventory (BICI), and Body Areas Satisfaction (BAS) subscales, and analyzed with Statistical Package for the Social Sciences (SPSS).

**Results:**

Women with Polycystic Ovary Syndrome reported significantly higher levels of depressive symptoms and body image concerns, along with lower body satisfaction, compared to those without Polycystic Ovary Syndrome. While a higher Body Mass Index (BMI) correlated with lower body satisfaction, no significant association was found between Body Mass Index and depressive symptoms.

**Conclusion:**

These findings highlight the psychological impact of Polycystic Ovary Syndrome on young women in the UAE, emphasizing the need for targeted mental health interventions and increased awareness.

## Introduction

Polycystic Ovary Syndrome (PCOS) is one of the most common syndromes affecting women of reproductive age [1], significantly affecting both physical and psychological well-being, often leading to depressive symptoms and lower body image satisfaction [2–4]. Recent studies highlight a growing prevalence of PCOS among women in the United Arab Emirates (UAE) [5,6]. However, the psychological effects of PCOS on women in the UAE remain underexplored, potentially due to cultural sensitivities surrounding reproductive health [7]. Menstruation is often viewed as a marker of femininity and reproductive health and can be disrupted by conditions like PCOS, leading to symptoms that include hirsutism (excessive hair growth), scalp hair loss, fertility issues, obesity, and acne, resulting in irregular periods, elevated androgen levels, and infertility in women [8,9]. These physical symptoms can contribute to psychological distress, including sleep disturbances and increased depressive symptoms [10–12]. For instance, Jedel et al. found that over 50% of the participants diagnosed with PCOS experienced sleep-related issues [10].

Given the rising prevalence of PCOS among women living in the UAE, this study aims to investigate its psychological effects, specifically comparing depressive symptoms and body image satisfaction between women with and without PCOS. Despite growing research on PCOS, a significant gap remains in understanding its impact on women in the UAE, where cultural standards and norms may hinder open discussion of female reproductive issues. Cultural factors in the UAE contribute to limited data on various aspects of women’s reproductive health, exacerbating disparities in awareness and care [13]. This gap is particularly evident among unmarried women, who are less likely to have regular access to reproductive health specialists and often lack knowledge about conditions like PCOS. In one study, only 21.7% of the sample population had sufficient awareness of the syndrome [14].

In the Middle East, topics related to reproductive and sexual health are considered culturally sensitive, which can limit public discussions and data collection. This sensitivity arises from cultural norms that prioritize privacy around personal health matters, especially those related to women’s reproductive health. Consequently, there is often a lack of comprehensive public health data on reproductive health, fertility, and related conditions like PCOS [15]. The limited availability of data can also be attributed to societal expectations that discourage open dialogue about women’s health issues, particularly among unmarried women. These cultural barriers may contribute to disparities among women in the UAE and a lack of awareness and understanding of conditions like PCOS, as well as infrequent visits to reproductive health specialists.

To reduce disparities in women’s health and mental health, education and awareness are essential. In Gulf Arab countries, cultural stigma around reproductive health creates significant barriers, as discussing these topics is often seen as taboo and inappropriate. While the healthcare system is advanced in the UAE, the understanding and the culture around PCOS and female reproductive system remains rare and needs awareness. This may delay diagnosis and lead to stigma. In addition, this stigma leads to misinformation and reluctance among young adolescent girls to visit reproductive health providers as they enter reproductive age [16]. Despite recommendations for girls to have their first gynecological visit between ages 13 and 15 [17], pelvic exams are typically deferred until sexual activity begins or until age 21. While girls do not need an exam to consult with a reproductive health specialist, the common misconception that women visit these specialists solely for sexual reasons further discourages them from seeking care. This stigma overlooks the importance of visits for preventive measures, such as screenings for endometriosis, PCOS, cervical cancer, infections, and even mental health concerns like Premenstrual Dysphoric Disorder (PMDD) and menstrual-related issues. The lack of proper education about women’s sexual health, combined with stigma surrounding both sexual and mental health, perpetuates disparities in the region [18].

### Objectives

This cross-sectional study focuses on the association between PCOS and psychological well-being, particularly body image satisfaction and depressive symptoms. The study uses the PHQ-9 to assess depression and validated scales to measure body image concerns. The independent variable is the presence of PCOS, while the dependent variables are depressive symptoms and body image satisfaction. By comparing these variables in women with and without PCOS, this research seeks to contribute to the existing literature and address the cultural and psychological challenges faced by women with PCOS in the UAE.

## Background: Polycystic ovary syndrome (PCOS) in the UAE

The increasing prevalence of polycystic ovary syndrome (PCOS) in the UAE underscores the importance of understanding its psychological impact. Studies have established a strong association between PCOS and various mental health issues, including depression, anxiety, and body image dissatisfaction [8,19–23]. Depressive symptoms in women with PCOS are often assessed using the PHQ-9, which measures low mood, fatigue, suicidal ideation, and other symptoms that align with the DSM-IV criteria for Major Depressive Disorder [24,25]. For a diagnosis, at least one of these symptoms must be either depressed mood or anhedonia, with a total of five or more symptoms required over a two-week period.

In addition to mental health concerns, body image dissatisfaction is a significant issue for women with PCOS. Body image is a multidimensional concept encompassing behaviors, thoughts, and evaluations of one’s physical appearance, including both positive and negative aspects [26]. Body image satisfaction refers to an individual’s assessment of their appearance, where dysmorphic appearance concerns, such as a preoccupation with perceived physical flaws, are increasingly recognized as a serious issue [27,28]. This preoccupation can lead to maladaptive behaviors like excessive checking or camouflaging [29,30]. The Body Image Concern Inventory (BICI) is a validated tool that measures these aspects and is particularly effective in classifying individuals with conditions like bulimia or Body Dysmorphic Disorder (BDD) [31].

Body Mass Index (BMI) is a critical factor in the assessment of women with PCOS, as it estimates body fat and helps determine their risk level for various diseases [32,33]. Research has found a correlation between PCOS and obesity, with a significant proportion of women with PCOS being either obese or overweight [34–37]. These weight issues further exacerbate the psychological burden of the condition, contributing to negative body image perceptions and mental health challenges [38].

Theories on body image, such as Social Comparison Theory and Objectification Theory, offer insights into the psychological mechanisms underlying these issues. Social Comparison Theory suggests that individuals evaluate themselves by comparing their appearance and behaviors to others, often leading to dissatisfaction [39]. Objectification Theory posits that societal emphasis on women’s looks fosters self-objectification, which can increase mental health risks [40,41]. Meanwhile, Self-Determination Theory emphasizes the importance of intrinsic motivation for psychological health, suggesting that a strong sense of self-determination may help mitigate the negative impacts of body image dissatisfaction [42,43].

Studies have consistently shown that women with PCOS are at a higher risk of experiencing depressive symptoms, body image dissatisfaction, and dysmorphic concerns, regardless of BMI [4,21,44]. Symptoms like hirsutism and obesity further intensify these negative perceptions, leading to a reduced quality of life [9]. Addressing these challenges requires comprehensive strategies, including weight management and hormonal treatments, which have been shown to improve both mental and physical health outcomes in women with PCOS [45].

## Methodological considerations

### Research questions

This research aims to answer one major question: What are the levels of depressive symptoms and body image satisfaction among young women in the UAE, and how does it differ between those diagnosed with Polycystic Ovary Syndrome (PCOS) and those without? To answer this question, the following sub-questions were formulated:

1. Do young women diagnosed with PCOS experience significantly higher levels of depressive symptoms compared to those without?
2. Do young women diagnosed with PCOS experience significantly lower levels of body image satisfaction compared to those without?
3. Do young women diagnosed with PCOS experience significantly higher levels of dysmorphic symptoms and appearance concerns compared to those without?
4. What is association between PCOS and BMI, Depressive Symptoms, Body Image Satisfaction, Dysmorphic Symptoms, and Appearance Concern?

### Methods

#### Procedure.

After receiving ethical approval from the Institutional Review Board (IRB), participants for the study were enlisted via social media platforms through a link to an online survey form developed on Microsoft Forms. The inclusion criteria, which were adherence to the specified age range and voluntary participation, were utilized in the participant selection process. Pregnant females, women under 18 and older than 35, and those incapable of granting informed consent constituted the exclusion criteria. Participant informed consent was obtained at the beginning of the survey, emphasizing the voluntary nature of the study, the participants’ right to withdraw at any time, the study’s objectives, and the confidentiality of their responses. Demographic information, including age, occupation, marital status, self-reported PCOS diagnosis, weight, and height, was subsequently requested following the respondent’s agreement to the informed consent. In the subsequent phase, participants completed the following assessments: the PHQ-9, containing nine items; the BICI, containing nineteen items; and finally, the MBSRQ-AS, BAS subscale, containing nine items [46].

Prior to participating in the study, all participants were provided with an informed consent form embedded within the survey. The consent form explained the purpose of the study, the voluntary nature of participation, and assurances of confidentiality and data protection. Participants were required to provide consent to proceed with the survey; otherwise, their responses could not be submitted. All participants included in the study provided written informed consent through an online survey platform.

The link to the survey was provided to the participants, who were granted the privacy and convenience of their preferred environment while completing the survey, thereby guaranteeing confidentiality. All participants provided electronic informed consent before participating. In addition, in order to uphold ethical principles and prevent any potential adverse effects on participants, ethical approval was obtained from the Institutional Review Board of the UAE University. The study received approval from the United Arab Emirates University Research Committee (Approval No. ERSC_2024_4138) on February 7, 2024. Participants were recruited from 2 March 2024 until 20 May 2024.

#### Research design.

This cross-sectional comparative study examined the association between depressive symptoms and body image satisfaction among young women in the UAE, comparing women with and without PCOS. Data were collected through a bilingual survey distributed to university students, their employers, social media users, and the participants’ relatives.

#### Data collection.

The independent variable was the presence or absence of PCOS, while the dependent variables included body image satisfaction and depressive symptoms. Participants self-reported their PCOS status. Power analysis was conducted to determine the appropriate sample size for the study. The G*Power 3.1 software was used to help determine the sample size and help minimize Type II errors. The following values were used to help determine the sample size.

The alpha level (α) was 0.05, the desired power (1-β) was 0.80, the statistical test was “Linear Bivariate Regression: Two Groups, difference between intercepts”, and the test family was t-test. Based on these factors, the power analysis determined that a minimum sample size of 57 participants in each group and a total of 114 was required to obtain adequate statistical power. This sample size assures that the study can identify a medium effect size with an 80% certainty, assuming a 5% significance level.

#### Participants.

The study included 221 young women aged 18 to 30 residing in the UAE. Convenience sampling recruited participants through universities and social media. Microsoft Forms link was distributed through university emails in the UAE, university student groups, and UAE-based social media groups. Weight and height were specifically collected to calculate the BMI. In addition, other demographic data such as age, occupation, and marital status were collected. Convenience sampling was chosen because of the cultural sensitivity and stigma associated with PCOS and mental health in the region, making it difficult to obtain a larger random sample.

Inclusion criteria included participants being female, willingness to participate, aged between 18 and 30, and residing in the UAE, regardless of nationality. Furthermore, participants were asked to respond with “yes” when inquired about ever receiving a diagnosis of Polycystic Ovary Syndrome (PCOS) from a medical professional. Exclusion criteria included pregnancy, age outside of the inclusion range, and inability to provide consent. A total of 30 women were excluded from the study for meeting the exclusion criteria.

#### Materials.

The survey included:

- **PHQ-9:** A 9-item tool to assess depressive symptoms [47]. Sun et al. [48] recorded a Cronbach’s alpha of α = 0.892 for the scale. An Arabic-translated version was also used in this study, demonstrating strong internal consistency with a Cronbach’s alpha of α = 0.857 [49,50].
- **Body Image Concern Inventory (BICI):** A 19-item questionnaire measuring dysmorphic appearance concerns [52], with high internal consistency α = 0.93 [53].
- **Multidimensional Body-Self Relations Questionnaire (MBSRQ-AS):** The Body Areas Satisfaction (BAS) subscale, a 9-item measure of body satisfaction [47,54]. With internal consistency coefficients ranging from α = 0.70 to 0.89 and 1-month test–retest reliabilities ranging from 0.74 to 0.91.

#### Reliability of translated psychometric scales.

Reliability data with Cronbach’s Alpha coefficients for translated instruments are included in Table 1. The BICI (19 items) and BAS (9 items) exhibit even better reliability at.970 and.901, respectively, while the PHQ (9 items) shows a respectable level of reliability at.885. Good internal consistency was demonstrated with 106 samples.

**Table 1:** Reliability Statistics.

	PHQ (9 items)	BICI (19 items)	BAS (9 items)	Overall (37 items)
Cronbach’s Alpha	.885	.970	.901	.872

n. = 106.

#### Procedure.

Ethical approval from the Institutional Review Board (IRB) of the UAE University was first obtained and participants were then recruited through social media platforms using a link to an online survey form developed with Microsoft. Inclusion criteria included adherence to the specified age range and voluntary participation, while exclusion criteria comprised pregnant women, those under 18 or over 35 years of age, and those unable to provide informed consent. Informed consent was obtained at the beginning of the survey, the right to withdraw at any time, the study’s objectives, and the confidentiality of responses. After consenting, participants provided demographic information, including age, occupation, marital status, self-reported PCOS diagnosis, weight, and height. They then completed the following assessments: the 9-item PHQ-9, the 19-item BICI, and the 9-item BAS subscale of the MBSRQ-AS. The survey was completed by participants in the privacy of their chosen environment to ensure confidentiality.

#### Data analysis plan.

Statistical analysis was conducted using IBM SPSS Statistics version 29.0.2.0. The main hypothesis— “young women in the UAE with PCOS have higher depressive symptoms and lower body image satisfaction compared to women without PCOS”—was tested using an independent t-test. Additionally, sub-hypotheses were examined, including whether women with PCOS have higher body image concerns, dysmorphic symptoms, appearance concerns, and BMI compared to women without PCOS. Correlations were also assessed to determine relationships between depressive symptoms, body image concerns, body area satisfaction, BMI, dysmorphic symptoms, and appearance concerns.

### Overview of the main findings

#### Demographic of participants.

Participants’ demographic variables are analyzed using frequencies and percentages. In the study, the occupation of the participants and total percentages for those with and without PCOS are shown in Table 2. Of individuals with self-reported diagnosis of PCOS, 43.6% are students, while 40.4% are employees, and 16.0% are other categories. Of those without PCOS, 43.3% are students, 46.5% are employed, and 10.2% are in other categories. Students and employees make up the majority of both categories overall, with a somewhat higher proportion of employees among those without PCOS. There are 221 people in the sample overall, 94 of whom have PCOS and 127 of whom do not. This approach is used to explain categorical data to provide a clear picture of the sample distribution.

**Table 2:** Demographic of Participants.

	With PCOS		Without PCOS		Overall	
	n.	%	n.	%	n.	%
**Occupation**						
Students	41	(43.6)	55	(43.3)	96	(43.4)
Employee	38	(40.4)	59	(46.5)	97	(43.9)
Other	15	(16.0)	13	(10.2)	28	(12.7)
**Marital Status**						
Single	79	(84.0)	104	(81.9)	183	(82.8)
Married	15	(16.0)	23	(18.1)	38	(17.2)
Total	94		127		221	

The data about the participants’ marital status is represented in Table 2 using percentages and frequencies. Table 2 shows overall percentages and the distribution of marital status for women with and without PCOS. Of the women with PCOS, 84.0% were single, while 16.0% were married. Similarly, women without PCOS, 81.9% were single, and 18.1% were married. Overall, 82.8% of the entire sample population were single whereas married women constitute for 17.2%.

#### Descriptive statistics of patient health questionnaire-9.

Descriptive statistics are used for each item in the PHQ-9 Questionnaire in Table 3, which include the mean, standard deviation, and percentage of the participants’ responses. According to the results in Table 3, the average overall score for the participants was 10.20 (SD = 6.23; range 0–27; N = 221).

**Table 3:** Descriptive Statistics of Patient Health Questionnaire-9.

Items			Percentages			
	Mean	SD	Not at all	Several Days	More than half the days	Nearly everyday
Item 1: “little interest or pleasure in doing things”	1.16	0.933	25.3	44.8	18.6	11.3
Item 2: “feeling down, depressed, or hopeless	1.12	0.907	25.8	46.6	17.6	10.0
Item 3: “trouble falling or staying asleep, or sleeping too much”	1.45	1.033	19.9	36.2	23.1	20.8
Item 4: “feeling tired or having little energy”	1.55	0.95	12.7	39.4	28.1	19.9
Item 5: “poor appetite or overeating”	1.39	0.969	19.5	37.6	27.6	15.4
Item 6: “feeling bad about yourself – or that you are a failure or have let yourself or your family down”	1.1	1.001	33.0	35.7	19.0	12.2
Item 7: “trouble concentrating on things, such as reading the newspaper or watching television”	1.21	1.05	30.8	33.5	19.9	15.8
Item 8: “moving or speaking so slowly that other people could have noticed? Or the opposite- being fidgety or restless that you have been moving around a lot more than usual”	0.74	0.949	52.9	28.1	10.9	8.1
Item 9: “thoughts that you would be better off dead, or of hurting yourself in some way”	0.48	0.861	70.1	18.1	5.4	6.3
Avg Mean Score	1.133	0.692				
Total	10.20	6.23				

Different depressive symptoms are represented by each item. For example, the mean score of 1.16 for Item 1 indicates that, on average, respondents have “little interest or pleasure in doing things”. The average total score for all items is given by the PHQ-9 Total, 10.20.

Understanding the association of depressive symptoms among women by these statistics provide information about their depressive symptoms. When investigating the mean scores of the participants with PCOS, they range between 0.48 for “item 9” and 1.55 for item 4 with standard deviation of 0.861 and 0.95 respectively. When investigating the percentages of responses, results show the response distribution range from “not at all” to “nearly every day”. For example, when checking item 1, it was found that 44.8% have “little interest or pleasure in doing things” several days in the past two weeks, while item 6, it was found that 19.0% of the participants was “feeling bad about yourself – or that you are a failure or have let yourself or your family down” more than half of the days in the past two weeks. On the other hand, 70.1% of the participants answered not at all for having “thoughts that you would be better off dead, or of hurting yourself in some way”.

#### Descriptive Statistics of Body Image Concern Inventory (BICI).

Descriptive statistics are used for calculating participant responses to the BICI items, which show a clear assessment of how many women from the sample have symptoms of body image concerns. The Body Image Concern Inventory (BICI) scores range from “never” to “always”.

According to Table 4, the total score of BICI items is 46.09 (SD = 20.87, range = 19–95) which indicates that participants on average report body image concerns between rarely and sometimes. On the other hand, when investigating the percentages of responses within the items, variations are found. For example, 34.4% of women answered never for item 6 which measures “my physical endurance is good”. Similarly, 34.8% answered never for item 4 “I like my looks just the way they are” indicating dissatisfaction with their appearance.

**Table 4:** Descriptive Statistics of BICI.

Items			Percentages				
	Mean	SD	Never	Rarely	Sometimes	Often	Always
Item 1: “before going out in public, I always notice how I look”	2.79	1.274	19.0	26.7	20.8	23.5	10.0
Item 2: “I am careful to buy clothes that will make me look at my best”	2.67	1.274	20.8	29.9	21.3	17.6	10.4
Item 3: “my body is sexually appealing”	2.14	1.19	38.5	29.0	17.6	9.5	5.4
Item 4: “I like my looks just the way they are”	2.39	1.319	34.8	21.7	21.7	12.7	9.0
Item 5: “I check my appearance on the mirror whenever I can”	2.59	1.368	29.4	22.2	20.4	16.3	11.8
Item 6: “my physical endurance is good”	2.45	1.366	34.4	22.2	17.2	16.3	10.0
Item 7: “most people would consider me good looking”	2.3	1.287	36.2	25.8	17.6	12.7	7.7
Item 8: “it is important that I always look good”	2.93	1.428	19.9	25.3	17.2	17.2	20.4
Item 9: “I use very few grooming products”	2.71	1.467	28.5	22.2	17.2	14	18.1
Item 10: “I like the way I look without my clothes on”	2.2	1.324	41.6	24.9	14	10.4	9
Item 11: “I am self-conscious if my grooming isn’t right”	2.6	1.409	31.7	19	19.5	17.2	12.7
Item 12: “I usually wear whatever is handy without caring how it looks”	2.66	1.401	28.5	21.3	20.4	15.8	14.0
Item 13: “I like the way my clothes fit me”	2.71	1.445	29.4	18.1	20.8	15.4	16.3
Item 14: “I don’t care what people think about my appearance”	2.65	1.336	25.8	23.1	24.0	14.9	12.2
Item 15: “I take special care with my hair grooming”	2.16	1.427	50.2	16.7	10.4	11.8	10.9
Item 16: “I dislike my physique”	1.9	1.211	54.8	19.5	12.7	7.7	5.4
Item 17: “I am physically unattractive”	2.22	1.344	44.8	17.2	17.6	12.2	8.1
Item 18: “I never think about my appearance”	2.02	1.277	50.7	19.0	14.5	9.0	6.8
Item 19: “I am always trying to improve my physical appearance”	2	1.323	53.4	19.5	9.5	9.5	8.1
Avg. Mean Score	2.24	1.09					
Total	46.09	20.87					
BICI Factors							
	Mean	SD					
Dysmorphic Symptoms (12 items)	31.25	13.84					
Appearance concern (7 items)	14.83	7.71					

Subscale scores are as follows: Dysmorphic Symptoms had a mean total score of 31.25 (SD = 13.84, range = 12–60), and Symptom Interference have a mean total score of 14.83 (SD = 7.71, range = 7–35). Higher BICI scores are associated with increased body image concern and interference. When investigating individual items, the mean score of 2.79 for Item 1 suggests that respondents often think about their appearance before going out in public. The distribution of responses across different levels of agreement is shown by the corresponding percentages. The BICI Total represents the average total score for all items, which in this case is 46.09. These figures provide valuable insights into body image perceptions and their potential impact on mental health and wellbeing by highlighting the degree of concern women have for their bodies.

#### Descriptive statistics of the body area satisfaction questionnaire.

In Table 5, descriptive statistics are used including means, standard deviations, and percentages across all items with a scale ranging from “Very Dissatisfied” to “Very Satisfied”. When investigating the participants responses, results show that the total score is 28.62 (SD = 8.35; range = 9–45) for all items. Lower scores indicate lower satisfaction with body areas, whereas higher scores reflect greater satisfaction. When investigating the individual items, some variations occur. For example, it was found that participants showed mostly dissatisfied for item 4 “mid torso (waist, stomach)” at 29.0%. Similarly, when investigating the percentages of other responses, some variations are found, for example, 27.1% of women were mostly dissatisfied for item 3 “lower torso (buttocks, hips, thighs, legs)”.

**Table 5:** Descriptive Statistics of the Body Area Satisfaction Questionnaire.

Items			Percentages				
	X	SD	Very Dissatisfied	Mostly Dissatisfied	Neither	Mostly satisfied	Very satisfied
Item 1: “face (features and complexations)”	3.71	1.12	3.2	14.9	17.6	36.7	27.6
Item 2: “hair (color, thickness, texture)”	3.4	1.238	7.7	18.6	22.6	28.5	22.6
Item 3: lower torso (buttocks, hips, thighs, legs)”	2.98	1.232	12.2	27.1	23.5	24.4	12.7
Item 4: “mid torso (waist, stomach)”	2.84	1.342	18.6	29.0	17.2	20.8	14.5
Item 5: “upper torso (chest or breasts, shoulders, arms)”	3.07	1.263	12.2	24.9	20.8	27.6	14.5
Item 6: “muscle tone”	3	1.238	11.8	27.6	23.5	23.5	13.6
Item 7: “weight”	2.62	1.304	23.5	31.7	13.6	22.2	9.0
Item 8: “height”	3.61	1.188	6.8	11.3	22.6	32.6	26.7
Item 9: “overall appearance”	3.40	1.126	6.3	16.7	23.1	38.5	15.4
Avg. Mean Score	3.179	0.927					
Total	28.62	8.35					

Scores for body image satisfaction shed light on women’s perceptions toward different areas of their bodies, which is crucial for understanding body image satisfaction and its possible effects on women. Findings demonstrate variant responses within each category in each scale. Lastly, in the BAS of the MSBRQ-AS, women with PCOS were neither satisfied nor dissatisfied with their body.

#### Independent samples T-tests between PCOS and Non-PCOS in depressive symptoms.

Independent samples t-test is used to compare and show whether depressive symptoms are higher in women with PCOS or women without PCOS. Table 6 presents the results of the t-tests which includes the means, standard deviations, and t-values for each item on the PHQ-9, with significant differences indicated (*p < 0.05, **p < 0.01). The mean total PHQ-9 score is greater for women with PCOS (M = 11.41, SD = 6.54) than for those without (M = 9.30, SD = 5.87). This difference is statistically significant (p < 0.05), showing that women with PCOS experienced considerably more depressed symptoms than those without PCOS. Significant variations are observed between the two groups for items 5, (“poor appetite or overeating”), item 7 (“trouble concentrating on things, such as reading the newspaper or watching television”), and the overall score. Results with p < 0.05 (*) and p < 0.01 (**) are considered statistically significant, indicating differences unlikely due to chance. These findings suggest that women with PCOS exhibit higher levels of depressive symptoms, particularly in areas like “feeling tired or having little energy” and “trouble concentrating on things”. While some items demonstrate significant differences, favouring higher depressive symptoms among women with PCOS, the overall score also reveals a significant difference between the two groups.

**Table 6:** Independent Samples T-tests between PCOS and Non-PCOS in Depressive Symptoms.

Items	Group				t.
With PCOS		Without PCOS		
	Mean	SD	Mean	SD	
Item 1: “little interest or pleasure in doing things”	1.30	.960	1.06	.902	1.907
Item 2: “feeling down, depressed, or hopeless	1.23	.955	1.03	.863	1.623
Item 3: “trouble falling or staying asleep, or sleeping too much”	1.60	.976	1.34	1.06	1.864
Item 4: “feeling tired or having little energy”	1.67	.872	1.46	.998	1.629
Item 5: “poor appetite or overeating”	1.55	.946	1.27	.971	2.193*
Item 6: “feeling bad about yourself – or that you are a failure or have let yourself or your family down”	1.24	1.054	1.00	.951	1.777
Item 7: “trouble concentrating on things, such as reading the newspaper or watching television”	1.45	1.043	1.03	1.023	2.950**
Item 8: “moving or speaking so slowly that other people could have noticed? or the opposite- being fidgety or restless that you have been moving around a lot more than usual”	.81	.942	.69	.955	.896
Item 9: “thoughts that you would be better off dead, or of hurting yourself in some way”	.56	.945	.42	.791	1.219
Total	11.4149	6.53594	9.2992	5.86666	2.484*

#### Independent samples T-tests of differences between PCOS and Non-PCOS in Body Image Satisfaction (Scale 1: BICI).

Independent samples t-test was used to compare BICI scores of women with and without PCOS. Items and total scores which showed significant differences between the groups were indicated by statistical significance of p < 0.05 (*) and p < 0.01 (**). An understanding of this aids the identification of body image satisfaction in both groups. Table 7 displays the results of independent samples t-tests assessing Body Image Satisfaction using the Body Image Concern Inventory (BICI) for women with and without PCOS. The table includes t-values, means, and standard deviations for each item. Results showed significant differences (p < 0.01) in the total mean score which is 51.17 for women with PCOS and 42.32 from women without. While investigating the individual items, significant differences were observed for several items, including item 4 “I like my looks just the way they are”, item 6 “my physical endurance is good”, item 11 “I am self-conscious if my grooming isn’t right”, item 12 “I usually wear whatever is handy without caring how it looks”, item 15, item 16, item 17, item 18 “I never think about my appearance”, item 19 “I am always trying to improve my physical appearance”, and the total. The overall mean score also shows a significant difference between women with and without PCOS. These findings suggest that women with PCOS generally have greater body image concerns compared to those without PCOS, with a marked difference in scores across individual items and in the total score.

**Table 7:** Independent Samples T-tests of Differences between PCOS and Non-PCOS in Body Image Satisfaction (Scale 1: BICI).

Items	Group				t.
With PCOS		Without PCOS		
	Mean	SD	Mean	SD	
Item 1: “before going out in public, I always notice how I look”	2.93	1.255	2.69	1.283	1.395
Item 2: “I am careful to buy clothes that will make me look at my best”	2.86	1.215	2.53	1.302	1.961
Item 3: “my body is sexually appealing”	2.31	1.192	2.02	1.178	1.766
Item 4: “I like my looks just the way they are”	2.71	1.325	2.16	1.269	3.136**
Item 5: “I check my appearance on the mirror whenever I can”	2.81	1.370	2.43	1.348	2.070*
Item 6: “my physical endurance is good”	2.79	1.436	2.20	1.262	3.137**
Item 7: “most people would consider me good looking”	2.50	1.326	2.15	1.241	1.995*
Item 8: “it is important that I always look good”	3.18	1.287	2.74	1.502	2.343*
Item 9: “I use very few grooming products”	2.95	1.394	2.54	1.500	2.099*
Item 10: “I like the way I look without my clothes on”	2.39	1.272	2.06	1.350	1.861
Item 11: “I am self-conscious if my grooming isn’t right”	2.90	1.415	2.38	1.368	2.773**
Item 12: “I usually wear whatever is handy without caring how it looks”	2.91	1.357	2.46	1.407	2.400*
Item 13: “I like the way my clothes fit me”	2.89	1.425	2.57	1.450	1.632
Item 14: “I don’t care what people think about my appearance”	2.89	1.372	2.46	1.284	2.362*
Item 15: “I take special care with my hair grooming”	2.71	1.514	1.76	1.213	5.044**
Item 16: “I dislike my physique”	2.22	1.361	1.65	1.026	3.405**
Item 17: “I am physically unattractive”	2.56	1.396	1.96	1.250	3.320**
Item 18: “I never think about my appearance”	2.31	1.336	1.81	1.193	2.862**
Item 19: “I am always trying to improve my physical appearance”	2.33	1.425	1.75	1.188	3.216**
Total	51.1702	21.23582	42.3228	19.85578	3.147**

#### Independent samples T-tests of differences between PCOS and Non-PCOS in Dysmorphic symptoms and appearance concern.

Independent samples t-tests were used to compare dysmorphic symptoms and appearance concern between women with and without PCOS. Results show significant differences (p < 0.05, p < 0.01) between both groups. Results in Table 8 show that the mean score of women with PCOS is 34.01 for dysmorphic symptoms and 17.15 for appearance concern, whereas it is 29.21 dysmorphic symptoms and 13.11 for appearance concern. For women without PCOS, this means that women with PCOS have greater levels of dysmorphic symptoms and appearance concerns as opposed to women without PCOS.

**Table 8:** Independent Samples T-tests of Differences between PCOS and Non-PCOS in Dysmorphic Symptoms.

Items	Group				t.
With PCOS		Without PCOS		
	Mean	SD	Mean	SD	
Dysmorphic Symptoms	34.0106	13.55554	29.2126	13.74116	2.586*
Appearance Concern	17.1596	8.30637	13.1102	6.77909	3.868**

#### Independent samples T-tests of differences between PCOS and Non-PCOS in body image satisfaction (Scale 2: MBSRQ-AS).

Independent samples *t*-tests were used to compare Body Area Satisfaction (BAS) scores between women with and without PCOS, as measured by the MBSRQ-AS. Table 9 shows the mean scores, standard deviations, and t-values for both groups, with significant differences indicated (*p < 0.05, **p < 0.01). Results show significant difference (p < 0.01) between the two groups, indicating that women with PCOS show less body area satisfaction. The mean score of women without PCOS is 30.4 compared to 26.1 for women with PCOS. This shows that women with PCOS report lower satisfaction with various body areas, including the face, hair, torso (lower, middle, and upper), muscle tone, weight, height, and overall appearance. These results suggest that women with PCOS generally have more negative body area perceptions compared to their non-PCOS counterparts.

**Table 9:** Independent Samples T-tests of Differences between PCOS and Non-PCOS in Body Image Satisfaction (Scale 2: BAS).

Items	Group				t.
With PCOS		Without PCOS		
	Mean	SD	Mean	SD	
Item 1: “face (features and complexations)”	3.44	1.232	3.91	.987	−3.041**
Item 2: “hair (color, thickness, texture)”	3.12	1.310	3.61	1.142	−2.896**
Item 3: lower torso (buttocks, hips, thighs, legs)”	2.78	1.237	3.13	1.211	−2.142*
Item 4: “mid torso (waist, stomach)”	2.60	1.322	3.02	1.333	−2.326*
Item 5: “upper torso (chest or breasts, shoulders, arms)”	2.79	1.243	3.28	1.240	−2.937**
Item 6: “muscle tone”	2.76	1.215	3.17	1.229	−2.516*
Item 7: “weight”	2.32	1.255	2.83	1.302	−2.972**
Item 8: “height”	3.32	1.220	3.83	1.120	−3.165**
Item 9: “overall appearance”	3.06	1.251	3.65	.956	−3.768**
Total	26.1702	9.01926	30.4252	7.34687	−3.746**

#### Independent samples T-tests of differences between PCOS and Non-PCOS in BMI.

In Table 10, results of independent sample t-tests reveal significant differences (p < 0.01) between women with PCOS and without. Women with PCOS have significantly higher mean score in BMI, with (mean = 27.239, SD = 6.1122) than those without PCOS (mean = 24.680, SD = 4.8834), suggesting that women with PCOS have higher body mass index, a common symptom of PCOS.

**Table 10:** Independent Samples T-tests of Differences between PCOS and Non-PCOS in BMI.

Items	Group				t.
With PCOS		Without PCOS		
	Mean	SD	Mean	SD	
BMI	27.239	6.1122	24.680	4.8834	3.341**

#### Two-tailed Pearson Corrections between BMI, Depressive Symptoms, Body Image Satisfaction, Dysmorphic Symptoms, and Appearance Concern.

In Table 11 the two-tailed Pearson correlations were used to examine the associations between Body Mass Index (BMI), Body Image Concern (BICI) total score, and depressive symptoms (PHQ-9 total). Significant correlations are indicated (p < 0.01) in most of the scales. See Table 11

**Table 11:** Two-tailed Pearson Corrections between BMI, depressive symptoms, and body image satisfaction.

	BMI	PHQ total	BICI total	BAS total
BMI	1	.129	.361^**^	−.371^**^
		.057	.000	.000
	220	220	220	220
PHQ total	.129	1	.506^**^	−.323^**^
	.057		.000	.000
	220	221	221	221
BICI Total	.361^**^	.506^**^	1	−.670^**^
	.000	.000		.000
	220	221	221	221
BAS Total	−.371^**^	−.323^**^	−.670^**^	1
	.000	.000	.000	
	220	221	221	221

**. Correlation is significant at the 0.01 level (2-tailed).

Findings reveal a positive correlation between BMI and depressive symptoms, though not significant. On the other hand, a significant positive correlation exists between BMI and body image concern. This means that the higher the BMI, the more body image concerns women with PCOS will have indicating a positive association between BMI and body image concerns. In contrary, BMI shows a significant negative correlation with body area satisfaction among women with PCOS. This suggests that higher BMI is associated with lower body area satisfaction in women with PCOS.

#### Percentage of depressive symptoms severity within PCOS and Non-PCOS.

Percentages of depressive symptom severity (none, mild, moderate, moderately severe, and severe) were calculated to show the distribution of depressive symptoms levels in both groups as shown in Table 12. Results show 34.0% of women with PCOS report experiencing moderate depressive symptoms, as opposed to 25.2% of women without PCOS. Furthermore, women with PCOS reported 28.7% mild symptoms, in contrast to 30.7% of women without PCOS. These findings suggest a correlation between PCOS and the intensity of depressive symptoms, indicating that women with PCOS experience higher levels of “Moderate” and “Severe” depressive symptoms compared to women without PCOS.

**Table 12:** Percentage of depressive symptoms severity within PCOS and Non-PCOS.

		Frequency	Percent
With PCOS	None	12	12.8
	Mild	27	28.7
	Moderate	32	34.0
	Moderately severe	11	11.7
	Severe	12	12.8
	Total	94	100.0
Without PCOS	None	32	25.2
	Mild	39	30.7
	Moderate	32	25.2
	Moderately severe	18	14.2
	Severe	6	4.7
	Total	127	100.0

## Discussion

In this study, the presence or absence of PCOS was examined as the independent variable and its correlations with depressive symptoms and body image satisfaction were analyzed. Participants were divided into two groups: women with PCOS (94 participants) and women without PCOS (127 participants). The findings indicate a positive correlation between PCOS and body image satisfaction. The disproportionate distribution between the two groups suggests a lower number of women with PCOS compared to those without. This imbalance may be attributed to cultural factors, such as cultural sensitivity or taboos, which could prevent women from seeking a clinical diagnosis or limit awareness of female reproductive disorders [19,20,47].

Our findings reveal that women with PCOS exhibited greater depressive symptoms and lower body image satisfaction compared to those without PCOS. This conclusion is supported by independent sample t-tests, which show that women with PCOS have a higher total score on the PHQ-9 (11.414) compared to women without PCOS (9.299). This result aligns with previous research by Cooney et al. [11], Simon et al. [12], and Jedel et al. [13], which indicates that women with PCOS experience more severe depressive symptoms. Lee and Dokras [14] also reported elevated depressive symptoms among women with PCOS, reinforcing our study’s findings. Xing et al. [15] similarly emphasized the biological and psychological mechanisms behind depression in PCOS. Despite the significant differences observed, some items on the PHQ-9, such as “poor appetite or overeating,” were notably higher in women with PCOS. This variation could be attributed to factors such as response biases in self-report surveys or cultural influences on mental health perceptions in the UAE. Andrade et al. [48] highlight the stigma surrounding mental health in the UAE, which may affect how participants report their symptoms. Cultural expectations and the societal portrayal of mental health could contribute to these discrepancies.

In examining body image satisfaction, the Body Image Concern Inventory (BICI) and the Body Area Satisfaction (BAS) scales were utilized. Our results indicate that women with PCOS have higher body image concerns and lower body satisfaction scores compared to women without PCOS. Women with PCOS scored 51.170 on the BICI, compared to 42.322 for those without PCOS, and 26.172 on the BAS, compared to 30.425 for women without PCOS. These findings align with Alur-Gupta et al. [49], Alkheyr et al. [16], and Barnard et al. [17], who also reported greater body image distress among women with PCOS. The BICI results indicate that women with PCOS are more concerned about their appearance, frequently checking their looks, and experiencing dissatisfaction with various body areas, such as their face, hair, and torso. Annagür et al. [18] note that symptoms such as acne and hair loss associated with PCOS may contribute to these concerns, while Bazarganipour et al. [8] further demonstrate the link between body image dissatisfaction and reduced self-esteem in PCOS women. The development of these scales is grounded in earlier body image research [50,51], supporting their use in this context.

Regarding dysmorphic symptoms and appearance concern, women with PCOS scored significantly higher (34.010 and 17.159, respectively) compared to those without PCOS (29.212 and 13.110, respectively). This supports Barberis et al. [21] and Schieber et al. [22], who observed that PCOS negatively impacts quality of life by exacerbating body dissatisfaction, dysmorphic concerns, and anxiety. BMI levels also differed between the groups, with women with PCOS having a mean BMI of 27.239 compared to 24.680 in women without PCOS. This finding aligns with Sadeeqa et al. [23], Pastore et al. [24], and Azizi Kutenaee et al. [25], indicating that obesity and poor sleep quality are common comorbidities in PCOS that worsen psychological outcomes [52,53].

Correlational analyses revealed no significant relationship between BMI and depressive symptoms. According to Pokora et al. [26], a higher BMI in PCOS is associated with greater concerns about body image and less satisfaction with body areas, although this does not always translate into higher depression symptoms. Depression may be triggered more by psychological and societal issues with body image than by weight itself [26,27]. Therefore, this study did not examine BMI as a primary predictor. However, depressive symptoms were positively correlated with body image concerns and negatively correlated with body area satisfaction. This suggests that women with PCOS experiencing higher depressive symptoms also report greater body image concerns and lower satisfaction with their body areas. These results are consistent with findings from Barber et al. [53], Himelein and Thatcher [54], and Berni et al. [28], which highlight the complex interplay between body image, neurodevelopmental vulnerabilities, and mental health in PCOS.

Our study also found that dysmorphic symptoms and appearance concerns positively correlate with depressive symptoms, BMI, and body image concerns, while negatively correlating with body area satisfaction. This underscores the multifaceted impact of PCOS on mental health and body image. The psychometric analysis of the PHQ-9, BICI, and BAS scales translated into Arabic showed good reliability, with Cronbach’s alpha values of.885,.970, and.901, respectively. This suggests that these tools are reliable for measuring depressive symptoms, body image concerns, and body area satisfaction in this population [29,30,54].

Our findings underscore the psychological impacts of PCOS on women in the UAE, as well as its prevalence. Notably, 81.9% of the women with PCOS in our sample were single. Zaitoun et al. [31] found that only 21.7% of the women studied had adequate education about PCOS. This highlights the significance of PCOS awareness and education, with most reproductive health efforts in the UAE focusing on married women, leaving unmarried women with limited knowledge, awareness, and education about their reproductive health and preventive strategies [32]. This contributes to the disparities faced by women in the UAE, with higher cases of PCOS and fewer preventive and intervention strategies targeted at the unmarried population.

Lifestyle factors such as diet, physical activity, and sleep may have acted as potential confounding variables, influencing both body image satisfaction and depressive symptoms. However, the use of validated tools, such as the PHQ-9 for depressive symptoms and the BAS and BICI scales for body image, supports the reliable interpretation of the observed associations in PCOS women. Psychological therapies such as Cognitive Behavioral Therapy [33] and Body Perception Treatment [34] have shown promise in addressing PCOS-related body image distress, while Self-Determination Theory [35] provides a framework for understanding how autonomy and intrinsic motivation may buffer negative outcomes. Theoretical perspectives such as Objectification Theory [36] and interoception-based models [37] further explain why body image dissatisfaction persists among women with PCOS. Future research should focus on integrating these frameworks into interventions to enhance the psychological health and general quality of life of this population.

The current literature on the psychological impact of PCOS predominantly originates from Western and non-Middle Eastern populations. This gap in research raises concerns about the generalizability of findings to women in Middle Eastern countries, particularly the UAE. The unique cultural, social, and economic factors present in the UAE may contribute to even higher levels of depressive symptoms and negative body image among women with PCOS compared to the populations studied in existing research. The scarcity of data specific to the UAE not only limits our understanding of the full extent of the psychological burden faced by these women but also underscores the urgent need for region-specific studies [47,48]. Addressing this gap could reveal a more accurate picture of the mental health challenges associated with PCOS in the UAE, guiding more effective interventions and support systems.

## Conclusions

There is an urgent need for impacted women in the UAE to receive proper psychological care. It is crucial to provide suitable treatments such as cognitive-behavioral therapy and psychosocial support services [33,34]. This study provides valuable insights into the psychological impact of PCOS among young women in the UAE, highlighting elevated depressive symptoms and lower body image satisfaction. The inclusion of diverse participants strengthens the study by providing a more comprehensive understanding of the psychological health of women across different segments of society. By addressing the reproductive health of women, the study highlights a significant need for improved awareness and education efforts in this area. The findings also underscore the need for appropriate psychological support and interventions, such as cognitive-behavioral therapy and psychosocial support services, for women with PCOS [8,27,49]. By examining the complex relationship between PCOS, depressive symptoms, and body image, this research contributes to the growing body of literature on PCOS and provides a foundation for future studies and clinical practices [17].

### Limitations

It is important to consider the limitations of this study. Self-report measures were used to obtain the data, which could lead to self-report bias. Nonetheless, self-reporting is a widely utilized method in research, especially when evaluating psychological constructs. Juber et al. [47] provided evidence in favor of using self-report methods in large-scale studies by showing a significant correlation between self-reported PCOS and clinical outcomes. In addition, BMI has been calculated using self-reported height and weight to validate the credibility of self-reported PCOS status, and the findings aligned with known clinical symptoms of the disorder. While self-reporting is often viewed as a limitation, the online survey format ensures confidentiality, which may help reduce social desirability bias, leading to more authentic responses.

Additionally, participant recruitment relied on distributing online surveys through universities and social media (in the UAE), which could be a limitation because the sample could have overrepresented younger women that are educated and socioeconomically advantaged women. Future studies can focus on expanding the recruitment methods to ensure a more representative sample.

### Research Implications

Prompt education and awareness among women in the UAE is imperative for reducing women’s reproductive and mental health disparities. The lack of information and awareness, compounded by societal taboos surrounding reproductive health, further deepens the health disparities among women. There is a need for public health initiatives that incorporate culturally appropriate and sensitive approaches to address these issues effectively. Understanding the correlation between PCOS, increased depressive symptoms, and lower body image satisfaction is important to develop interventions that effectively target the needs of young women in the UAE. This research highlights the need for more studies on the psychological effects of PCOS.

Future investigations should concentrate on long-term cohort studies to look at the progression of psychiatric disorders in women with diagnosed PCOS over time and pinpoint potential risk and protective factors linked to mental health consequences. Conducting longitudinal studies will provide a more profound understanding of how mental health issues can change over time and enable us to identify factors that may either exacerbate or alleviate symptoms. This research avenues for developing personalized interventions tailored to meet the evolving needs of women with PCOS as they navigate through different life stages.

Furthermore, research examining the cultural and societal elements impacting the perception of body image and the stigma associated with mental health in the UAE is necessary to create culturally competent therapies that meet the requirements of the community. Recognizing these factors can enhance awareness and understanding among both men and women, promote education, and foster greater empathy towards women experiencing these issues.

Future research could focus on understanding PCOS more by investigating the lifestyle factors and stress levels of women in the UAE which could also provide insights into potential risk factors contributing to the rising prevalence of PCOS. Exploring demographic variations across different emirates and economic backgrounds will help determine if these factors influence PCOS diagnosis and provide a more nuanced understanding of its impact. Comparing PCOS manifestations in women across different age groups could reveal how PCOS evolves throughout various life stages. Future studies should also explore the relationship between PCOS and other health conditions, such as diabetes and hypertension, to better understand the co-occurrence of these conditions and improve management strategies. For example, research by Ho et al. [54] found that women with Hashimoto’s syndrome have a 2.37-fold increased likelihood of developing PCOS, highlighting the need for further investigation into the interplay between these conditions.

## Supporting information

S1 Dataset. (XLSX)
